# Detecting CSSLs and yield QTLs with additive, epistatic and QTL×environment interaction effects from *Oryza sativa* × *O. nivara* IRGC81832 cross

**DOI:** 10.1038/s41598-020-64300-0

**Published:** 2020-05-08

**Authors:** Divya Balakrishnan, Malathi Surapaneni, Venkateswara Rao Yadavalli, Krishnam Raju Addanki, Sukumar Mesapogu, Kavitha Beerelli, Sarla Neelamraju

**Affiliations:** ICAR-Indian Institute of Rice Research, Hyderabad, India

**Keywords:** Plant breeding, Plant hybridization, Plant molecular biology

## Abstract

Chromosome segment substitution lines (CSSLs) are useful tools for precise mapping of quantitative trait loci (QTLs) and the evaluation of gene action and interaction in inter-specific crosses. In this study, a set of 90 back cross lines at BC_2_F_8_ generation derived from Swarna x *Oryza nivara* IRGC81832 was evaluated for yield traits under irrigated conditions in wet seasons of 3 consecutive years. We identified a set of 70 chromosome segment substitution lines, using genotyping data from 140 SSR markers covering 94.4% of *O. nivara* genome. Among these, 23 CSSLs were significantly different for 7 traits. 22 QTLs were detected for 11 traits with 6.51 to 46.77% phenotypic variation in 90 BILs. Three pleiotropic genomic regions associated with yield traits were mapped on chromosomes 1, 8 and 11. The marker interval RM206-RM144 at chromosome 11 was recurrently detected for various yield traits. Ten QTLs were identified consistently in the three consecutive years of testing. Seventeen pairs of significant epistatic QTLs (E-QTLs) were detected for days to flowering, days to maturity and plant height. Chromosome segments from *O. nivara* contributed trait enhancing alleles. The significantly improved lines and the stable QTLs identified in this study are valuable resource for gene discovery and yield improvement.

## Introduction

Rice is one of the major crops which grow across wide range of environments and supports majority of the world population mainly located in developing countries of Asia, West Africa, Latin America and the Caribbean. Global rice yields and total rice consumption are projected to rise by 12% and 13% respectively by 2027^1^. However there is a large gap between anticipated production and consumption due to expected population rise and restricted cultivated area expansion in major rice growing countries of Asia. Even though a large number of rice cultivars exist in the world, the most widely grown varieties are few and they harbour a limited genetic diversity due to common parents and origin within *Oryza sativa* species. The crop is exposed to multiple stresses and evolving pests, as range of cultivation is wide and also climate scenario is changing. Therefore, much focus is needed to widen the gene pool of cultivated germplasm to sustain and improve yield potential. Prebreeding and utilization of wild accessions for introgression of novel genes / QTLs / chromosomal regions are gaining more importance in rice improvement^2,3^.

Chromosome segment substitution lines (CSSLs) are prebreeding genetic stocks which can be utilised in crop breeding and genomics studies for improvement of various traits^4^. Wild species are a reservoir of valuable genes, however the unpredictability of genotypic potential of wild species in a cultivar background, cross incompatibility and sterility in inter specific crosses are common hindrance in prebreeding programs. CSSLs developed using wild donors can overcome these problems as a bridging prebreeding product and can be used for identification, mapping and introgression of novel alleles from the wild. CSSLs can be utilized for faster and easier development of NILs and varieties than using an un-adapted or wild genotype directly. Since a single major introgression is present in each line, its effects are not masked by other components of donor genome and thus linkage drag and negative effect on QTL is limited in an ideal CSSL set^4,5^.

About fifty inter-specific and intra-specific CSSL libraries are reported in rice in the last two decades. Most of the initial CSSLs developed in rice involved *indica*/ *japonica* crosses but the use of wild species as donor has increased since the last two decades. Accessions from wild rice species *viz*., *O. rufipogon*, *O. nivara, O. glumaepatula*, *O.barthii*, and *O. meridionalis* were used as donor parent in CSSL development programmes^6–8^. Furuta et al^9^. developed CSSLs using *Oryza rufipogon* accessions as donor. Rangel et al^10^. developed CSSL in *indica* cultivar BG 90–2 using *O. glumaepatula*. Arbelaz et al^11^. developed two populations of interspecific introgression lines (ILs) in *cv*. Curinga, a tropical *japonica* upland cultivar using two wild donor species, *O.meridionalis* and *O. rufipogon* Griff having 76.73% to 97.6% donor genome respectively. CSSLs are ideal for accurate mapping of gene/ QTL and genetic analysis, largely due to low interaction of genetic backgrounds^12^. Thus, CSSLs have been used to identify chromosome regions affecting any trait^13^, mapping, fine-mapping and cloning genes from novel genomic regions and QTL hotspots^14^. In rice, superiority of introgression lines over recurrent parents has been reported^15–22^, however the effect of alien introgression using CSSLs and their advantage over RILs/ BILs has not been studied extensively. Ideally if each CSSL has only a segment from the donor it becomes easy to define the QTL position. Usually a primary CSSL population contains more than one donor segment in each line, so the gene and marker frequencies do not fit into mendelian ratios as that of F_2_ and BC mapping population^23^. Therefore, stepwise regression-based likelihood ratio test (RSTEP-LRT) is used for QTL mapping in CSSLs. Inclusive composite interval mapping (ICIM)^24,25^ was used in QTL mapping and to study the epigenetic interactions due to its unbiased QTL effect estimation^26,27^.

In our previous study, population was developed from Swarna /*O. nivara* IRGC81848 for QTL mapping for yield traits. Individually advanced lines were genotyped at BC_2_F_8_ and a set of 74 CSSLs were constructed with donor genome coverage of 89%^20^. Another population developed from a cross between Swarna and *O.nivara* IRGC81832, was evaluated for yield and quality related traits and QTLs were mapped at BC_2_F_2_ generation^16,21,28^. This population was advanced to BC_2_F_8_ and was phenotyped in this study for 3 consecutive wet seasons and genotyped using SSR markers to identify a set of CSSLs representing complete genome of *O. nivara* as overlapping segments substituting Swarna segments and to detect major QTLs for yield traits. Phenotyping in multi environments is essential to determine stable constitutive QTLs as well as environment specific adaptive QTLs. The mapping population was tested for 3 years for identifying environment and epistatic interactions of QTLs using multiple mapping procedures to identify stable significant QTLs and to understand the genetic implications of these QTLs.

## Methods

The research work was conducted at Indian Institute of Rice Research (IIRR), Hyderabad during the period 2014 to 2017 wet seasons. The area is located at latitude of 11°00′ N longitude of 77°00′ E and an elevation of 426.72 m above mean sea level (MSL).

### Plant material

Advanced back cross introgression lines derived from a cross between an elite cultivar Swarna (*O. sativa*) as a recurrent parent and a wild accession *O. nivara* IRGC81832 as a donor parent^16^ were used in this study at BC_2_F_8_ to BC_2_F_10_ stage after conducting single panicle selection for consecutive generations from BC_2_F_5_ and these NPK or K lines are now designated as NK 1–90.

### Phenotypic evaluation

The experiment was conducted following randomised complete block design (RCBD) with three replications at experimental plots in Indian Institute of Rice Research (IIRR), Hyderabad. A set of advanced backcross introgression lines (BC_2_F_8_) along with recurrent parent Swarna, were raised during wet seasons of 2014 (E1), 2015 (E2) and 2016 (E3). 25 days old seedlings were transplanted following 15 cm interplant spacing and 20 cm inter row spacing. Package of practices for fertilizer application and pest control measures under irrigated conditions were followed to avoid yield loss. Randomly selected five plants from each replication were evaluated for 9 yield- related traits, *viz*., days to 50% flowering (DFF), days to maturity (DM), plant height (PH), number of tillers per plant (NT), number of productive tillers per plant (NPT), biomass (BM), single panicle weight (PW), grain yield/plant (GY), bulk yield per square metre (BY) and two derived traits; harvest index (HI) and per day productivity (PDP). The observations on yield and morpho-agronomic traits were recorded from the field experiments following Standard Evaluation System (IRRI, 2013).

The phenotypic data was analysed using Statistical Tool for Agricultural Research (STAR v2.0.1, IRRI) for the descriptive statistics (Supplementary Table 1) and ANOVA (Supplementary Table 2). Broad-sense heritability was calculated using the formula: h^2^ = σg^2^/(σg^2^ + σe^2^), where σ^2^g is the genotypic variance and σ^2^e is the environmental variance^29^. Levene and Bartlett test for homogeneity of variance was conducted for yearly means and a Shapiro-Wilk test for normality (Supplementary Table 3 and 4) were performed to test the assumptions of the ANOVA (Supplementary Table 5). Significant pairwise comparisons were carried out comparing with control Swarna. Boxplot and histogram of each trait in different seasons were derived using R software^30^. Phenotypic and genotypic correlation among trait averages was computed for Prob > |r| at 0.05, 0.01 and 0.001 in STAR (STAR v2.0, IRRI) using Pearson’s product-moment correlation using replicated phenotypic data across the years. The observed trait data were analyzed with PB tools (Version 1.4, http://bbi.irri.org/products) by considering blocks as random and entry as fixed effect to obtain LS mean estimates and were then used for QTL analysis.

### SSR genotyping

Genomic DNA was extracted from leaf using CTAB method. The extracted DNA was estimated at 260 nm/280 nm and 260 nm/230 nm for quality and quantity using UV spectrophotometer. The estimated DNA quantity was between 94 to 1294 ng/ul with 1.8 to 2.1 OD at 260 nm/280 nm and 260 nm/230 nm. For genotyping, working DNA concentration of 40 ng/ul was made as template. A set of 300 markers including 165 SSRs^31^ which are equally distributed throughout rice genome were used to study parental polymorphism and 140 SSR markers, identified as polymorphic were used in screening CSSL population. Genomic DNA was isolated from fresh leaf samples of 90 lines following CTAB method. 140 SSR makers distributed across the genome were used to genotype the mapping population. PCR was performed in thermal cycler (G-STORM, USA) with a final reaction volume of 10 µl containing 15 ng of genomic DNA, 1X assay buffer, 200 μM of dNTPs, 1.5 mM MgCl_2_, 10 pmol of forward and reverse primer and 1 unit of Taq DNA polymerase (Thermo Scientific). PCR cycles were programmed as follows: initial denaturation at 94 °C for 5 min followed by 35 cycles of 94 °C for 30 sec, 55 °C for 30 sec, 72 °C for 1 min and a final extension of 10 min at 72 °C. Amplified products were resolved in 3% agarose gel prepared in 0.5 X TBE buffer and electrophoresis was carried out at 120 V for 2 h. Gels were stained with ethidium bromide and documented using gel documentation system (Alfa imager, USA).

### Identification of CSSL and QTL analysis

SSRs were scored as co-dominant markers, and recorded as “A” for band presence female parent Swarna and “B” for presence of male *O.nivara* parent and “AB” for heterozygous bands. CSSLs were identified using segregation data of 140 polymorphic SSR markers in 90 BILs in the background of Swarna genome using CSSL Finder (http://mapdisto.free.fr/CSSLFinder/). Substituted chromosome segments were estimated on the basis of origin of alleles of flanking markers. A chi-square test for the 1:1 segregation ratio was conducted in the advanced backcross mapping population and no segregation distortion was found. Linkage map was constructed based on genotypic data of 90 BILs using 140 polymorphic SSR markers on all chromosomes. MAP functionality was used for the construction of genetic linkage map involving interfaces of grouping, ordering, and rippling procedures in map construction. Ordering of loci is done by an efficient approximate algorithm of nnTwoOpt in ICIM.

The Kosambi’s mapping function was used to transform recombination frequencies between linked loci into cM distances. QTL mapping was performed by single marker analysis (SMA), inclusive composite interval mapping (ICIM-ADD) of additive and dominant QTL and inclusive composite interval mapping of epistatic QTL (ICIM-EPI) functions implemented in the QTL IciMapping v4.1 (www.isbreeding.net) in a stepwise regression for the adjusted means of each trait. The threshold LOD value was determined by a permutation test involving 10000 runs at a significance level of p = 0.05 and the QTLs in a particular genomic region with LOD values larger than this threshold for each trait were used. The LOD test statistic used was −2ln (L0/L1), where L0/L1 is the ratio of the likelihood under the null hypothesis (there is no QTL in the interval) and the alternative hypothesis (there is a QTL in the interval). The QTLs were deemed to exist only at positions where the LOD score exceeded the corresponding significant threshold. Estimation of position, genetic effects and phenotypic variation percentage of QTLs was done at the significant LOD peak in the region under consideration. The phenotypic variance explained by a single QTL was estimated by the square of the partial correlation coefficient (R^2^). Estimates of the R^2^ value or the phenotypic variance explained (PVE) and the additive effects of QTL at their peak LOD positions were obtained from the output of QTL analysis. Parent for trait enhancing allele was identified using the sign of the additive effects, negative sign denotes that trait -enhancing allele is from *O. nivara*.

Mapping of additive and digenic epistasis genes with chromosome segment substitution lines was carried out with QTL IciMapping v4.0^26^ with CSL (Chromosome Segment Line) option using method ‘Likelihood ratio test based on stepwise regression for additive QTL’ (RSTEP-LRT-ADD) which specifically deals with QTL mapping in collections with multiple introgressions per line^32^. Parameters setting was ‘Multi collinearity control’= −1 (equivalent to deletion of duplicated markers only) and ‘PIN’ = 0.0001 (PIN: the largest-value for entering variables in stepwise regression of residual phenotype on marker variables). Logarithm of odds (LOD) threshold values for each trait was computed after 10000 permutations using a type I error=0.05 and reported in corresponding tables as footnotes. The proportion of variance explained by a single QTL was as provided from single marker analysis in RSTEP-LRT-ADD. QTL-by-environment interaction analysis in biparental population was carried out using MET. Epistatic effect QTLs were analysed using ICIM-EPI with a probability value (PIN) of 0.0001 and threshold LOD^26^.

## Results

### Phenotyping

The variance components and descriptive statistics of yield related traits are presented in Table 1. The genotypes showed significant variation for BY, PH, DFF, DM, BM and TDM in the BIL population. High broad-sense heritability (h^2^) of 96.43%, 95.98%, 94.15% and 58.45% was observed for days to flowering, days to maturity, plant height and panicle weight respectively. The variance component associated with genotypes contributed to 92% of total variation. The mean performance of BILs using pooled data for three seasons showed significant increase over Swarna for 7 traits. 31 transgressive BILs that were significantly superior or inferior to the recurrent parent were identified after evaluating the BILs for yield traits over three years from their frequency distribution (Supplementary Fig. 1). Of these BILs, NK61 out-performed Swarna for GY and NK58 and NK83 for BY in all three seasons. 18 BILs with significantly lower DFF than Swarna were identified. For PH and BM, 13 and 7 BILs were identified respectively with significantly higher trait values compared to Swarna (Supplementary Table 6).

**Table 1:** Variability, descriptive statistics and yield traits means (±standard deviation) of parents and of BC_2_F_8_ population.

Trait	Parents		Mapping Population															Heritability
	Swarna	*Oryza nivara* (IRGC81832)	Swarna / *Oryza nivara* (IRGC81832) BC_2_F_8_															
	(mean ± SE)		(mean ± SE)		(mean ± SE)		Variance		Skewness		Kurtosis	Minimum	Maximum	Range	W-test	P-value	P-value	
DFF	120 ± 4.3		132 ± 7.9		112.95 ± 6.36		40.51		−2.01		5.2	83	121	38	0.82	0.00E + 00	0.00E + 00	0.97
DM	154 ± 2.8		157 ± 5.2		142.27 ± 6.37		40.61		−1.93		4.95	112.5	151	38.5	0.84	0.00E + 00	0.00E + 00	0.96
PH	86 ± 3.2		124 ± 5.3		84.31 ± 19.13		365.83		1.25		0.64	60.44	137.22	76.78	0.84	0.00E + 00	0.00E + 00	0.97
NT	12 ± 2.0		58 ± 6.0		12.05 ± 1.56		2.42		0.94		2.49	8.44	17.89	9.45	0.94	1.20E-03	1.20E-03	0.32
NPT	11 ± 1.6		48 ± 5.0		10.7 ± 1.48		2.2		0.46		0.9	6.89	15.44	8.55	0.98	5.64E-01	5.64E-01	0.28
PW	1.83 + 0.15		0.93 + 0.03		2.08 ± 0.46		0.21		0.3		−0.66	1.11	3.15	2.04	0.96	5.32E-02	5.32E-02	0.80
GY	12 ± 1.4		1.80 + 0.47		13.19 ± 3.91		15.32		0.86		1.38	5.23	27.73	22.5	0.96	2.16E-02	2.16E-02	0.76
BM	11 ± 1		16.00 + 2.65		25.96 ± 6.68		44.63		0.78		0.3	13.77	44.64	30.87	0.94	1.33E-03	1.33E-03	0.81
BY	—		—		418.46 ± 98.62		9726.42		1.06		1.38	265.8	737.97	472.17	0.92	1.14E-05	1.14E-05	0.66
TDM	13 ± 2		17.80 + 2.74		39.15 ± 9.43		88.89		0.74		0.25	22.27	65.95	43.68	0.95	2.17E-03	2.17E-03	0.79
HI	34.47 ± 6.73		10.50 + 3.02		33.37 ± 5.88		34.61		−0.01		−0.07	16.36	46.62	30.26	0.99	8.08E-01	8.08E-01	0.78
PDP	—		—		0.09 ± 0.03		0.82		1.06		2.01	0.04	0.21	0.17	0.93	1.34E-04	1.34E-04	0.78
	**G Mean**			**PCV**			**GCV**			**h**^**2**^			**GA**			**GG**		
	**2014**	**2015**	**2016**	**2014**	**2015**	**2016**	**2014**	**2015**	**2016**	**2014**	**2015**	**2016**	**2014**	**2015**	**2016**	**2014**	**2015**	**2016**
DFF	110.33	115.53	112.98	5.21	6.54	5.89	5.21	6.54	5.56	99.99	99.99	89.30	11.83	15.57	12.23	10.72	13.48	10.83
DM	139.02	145.51	142.26	4.23	5.19	4.67	4.23	5.19	4.38	100.00	99.86	88.07	12.11	15.55	12.05	8.71	10.69	8.47
PH	83.97	85.05	83.90	27.81	21.24	25.78	26.92	20.67	25.00	93.73	94.71	94.01	45.09	35.25	41.89	53.70	41.44	49.93
NT	11.86	11.46	12.83	40.23	25.89	28.46	9.80	12.31	15.05	5.94	22.60	27.96	0.58	1.38	2.10	4.92	12.05	16.39
NPT	11.20	9.49	11.41	40.09	33.57	28.85	10.29	16.41	13.23	6.58	23.89	21.04	0.61	1.57	1.43	5.44	16.52	12.50
PW	2.32	1.85	2.07	35.09	32.79	33.52	23.23	24.93	28.79	43.80	57.80	73.76	0.73	0.72	1.06	31.66	39.04	50.94
GY	13.15	13.48	12.95	58.77	47.66	44.54	36.14	30.86	32.76	37.82	41.94	54.09	6.02	5.55	6.43	45.79	41.17	49.63
BM	24.63	28.47	24.78	49.00	37.68	32.80	28.45	23.89	27.12	33.72	40.19	68.39	8.38	8.88	11.45	34.03	31.20	46.20
BY	461.76	405.27	388.35	43.52	42.38	44.57	22.40	27.35	30.75	26.48	41.66	47.59	109.63	147.40	169.69	23.74	36.37	43.69
TDM	37.78	41.95	37.73	48.02	36.78	30.47	26.42	23.19	25.48	30.27	39.76	69.95	11.31	12.63	16.56	29.94	30.12	43.90
HI	34.04	31.99	34.08	32.69	26.11	28.10	25.43	16.81	19.41	60.50	41.47	47.74	13.87	7.14	9.41	40.75	22.30	27.63
PDP	0.10	0.09	0.09	60.17	48.79	46.03	37.90	32.57	34.38	39.66	44.57	55.78	0.05	0.04	0.05	49.16	44.79	52.89

DFF- days to 50% flowering, DM- days to maturity, PH- plant height, NT- number of tillers, NPT- number of productive tillers, PW- panicle weight, GY- yield per plant, BM- Biomass, BY- bulk yield, TDM-total dry matter, HI- harvest index, PDP- per day productivity, G Mean- Genotypic mean, PCV-Phenotypic coefficient of variation, GCV-Genotypic coefficient of variation, h^2^-Heritability, GA-Genetic advance, GG- Genetic gain.

Among 31 lines identified through significant pair wise comparison 21 showed significantly positive values for 7 traits and 16 showed significantly negative values for 3 traits compared to Swarna. Highly significant phenotypic associations were observed among the traits and across years (Table 2). Although all yield traits were positively associated and were significant at P < 0.01, DFF and DM showed negative association with all yield traits except biomass. GY showed strong positive correlation (P < 0.01) with NT, NPT, BM, PW, TDM, HI and PDP. PH was highly significantly correlated with both TDM and BM. GY showed highly significant correlation with BM and BY. PW was significantly correlated with GY, TDM, HI, BY and PDP but negatively with DFF and DM.

**Table 2:** Phenotypic and genotypic correlation coefficients among twelve yield related traits for three environments.

		DFF	DM	PH	NT	NPT	PW	GY	BM	BY	TDM	HI	PDP
**DFF**	**Mean**	1	0.997**	−0.116	0.159	0.197	−0.331**	−0.263**	0.230**	−0.295**	0.059	−0.507	−0.426**
	2014												
	2015												
	2016												
**DM**	**Mean**	0.992**	1	−0.125	0.151	0.186	−0.349**	−0.279**	0.209	−0.304**	0.037	−0.506**	−0.438**
	2014	0.989**											
	2015	0.999**											
	2016	0.989**											
**PH**	**Mean**	−0.069	−0.073	1	0.076	0.160	0.464**	0.405**	0.816**	0.469**	0.750**	−0.299**	0.384**
	2014	−0.180*	−0.189*										
	2015	0.065	0.064										
	2016	−0.124	−0.134										
**NT**	**Mean**	−0.002	−0.005	0.032	1	0.991**	−0.036	0.316**	0.326**	0.435**	0.363**	−0.005	0.278**
	2014	0.124	0.115	0.000									
	2015	−0.160	−0.161	−0.023									
	2016	0.044	0.057	0.127									
**NPT**	**Mean**	−0.018	−0.032	0.058	0.893**	1	−0.086	0.322**	0.464**	0.259**	0.438**	−0.077	0.137
	2014	0.107	0.096	0.009	0.976**								
	2015	−0.020	−0.022	0.118	0.689**								
	2016	0.031	0.039	0.110	0.928**								
**PW**	**Mean**	−0.264**	−0.285**	0.243**	0.039	0.077	1	0.833**	0.489**	0.819**	0.689**	0.428**	0.839**
	2014	−0.273**	−0.282**	0.260**	0.026	0.034							
	2015	−0.295**	−0.297**	0.148	0.015	0.003							
	2016	−0.055	−0.073	0.343**	0.014	0.031							
**GY**	**Mean**	−0.125	−0.13	0.188*	0.249**	0.268**	0.391**	1	0.550**	0.989**	0.801**	0.546**	0.984**
	2014	−0.110	−0.133	0.101	0.469**	0.501**	0.464**						
	2015	−0.169*	−0.168*	0.189*	0.027	0.092	0.326**						
	2016	−0.140	−0.148	0.319**	0.077	0.072	0.399**						
**BM**	**Mean**	0.15	0.149	0.437**	0.289***	0.279**	0.215*	0.559**	1	0.634**	0.941**	−0.370**	0.464**
	2014	0.111	0.102	0.349**	0.514**	0.515**	0.305**	0.661**					
	2015	0.141	0.140	0.438**	0.108	0.171*	0.115	0.591**					
	2016	0.095	0.074	0.614**	0.198*	0.195*	0.384**	0.349**					
**BY**	**Mean**	−0.137	−0.144	0.192*	0.145	0.184*	0.357**	0.756**	0.352**	1	0.868**	0.465**	0.986**
	2014	−0.098	−0.109	0.122	0.259**	0.292**	0.320**	0.625**	0.383**				
	2015	−0.184	−0.188*	0.194*	0.002	0.086	0.299**	0.828**	0.439**				
	2016	−0.039	−0.030	0.293**	0.116	0.092	0.384**	0.911**	0.289**				
**TDM**	**Mean**	0.049	0.046	0.383**	0.307**	0.309**	0.319**	0.821**	0.932**	0.572**	1	−0.044	0.732**
	2014	0.026	0.011	0.275**	0.542**	0.556**	0.401**	0.867**	0.947**	0.521**			
	2015	0.028	0.028	0.384**	0.086	0.157*	0.216*	0.827**	0.942**	0.650**			
	2016	−0.003	−0.022	0.594**	0.178*	0.174*	0.472**	0.749**	0.882**	0.662**			
**HI**	**Mean**	−0.262**	−0.264**	−0.15	0.02	0.053	0.223*	0.595**	−0.252**	0.505**	0.086**	1	0.612**
	2014	−0.246**	−0.263**	−0.191*	0.089	0.130	0.264**	0.563**	−0.146	0.399**	0.144		
	2015	−0.323**	−0.321**	−0.156*	−0.089	−0.063	0.274**	0.561**	−0.269**	0.502**	0.046		
	2016	−0.216*	−0.207*	−0.089	−0.083	−0.076	0.082	0.699**	−0.371**	0.654**	0.088		
**PDP**	**Mean**	−0.226*	−0.232*	0.186*	0.245**	0.266**	0.413**	0.992**	0.525**	0.751**	0.794**	0.612**	1
	2014	−0.190*	−0.211*	0.110	0.449**	0.482**	0.478**	0.995**	0.638**	0.623**	0.849**	0.577**	
	2015	−0.280**	−0.279**	0.178*	0.044	0.093	0.356**	0.990**	0.549**	0.827**	0.794**	0.584**	
	2016	−0.246**	−0.255**	0.314**	0.065	0.059	0.391**	0.990**	0.322**	0.889**	0.725**	0.707**	

Significance levels: *P < 0.05, and **P < 0.01. Upper diagonal shows the genotypic correlation of mean data across years and lower diagonal shows phenotypic correlations of year wise and mean data. DFF- days to 50% flowering, DM- days to maturity, PH- plant height, NT- number of tillers, NPT- number of productive tillers, PW- panicle weight, GY- yield per plant, BM- Biomass, BY- bulk yield, TDM-total dry matter, HI- harvest index, PDP- per  day productivity.

### CSSL identification

**Figure 1:**
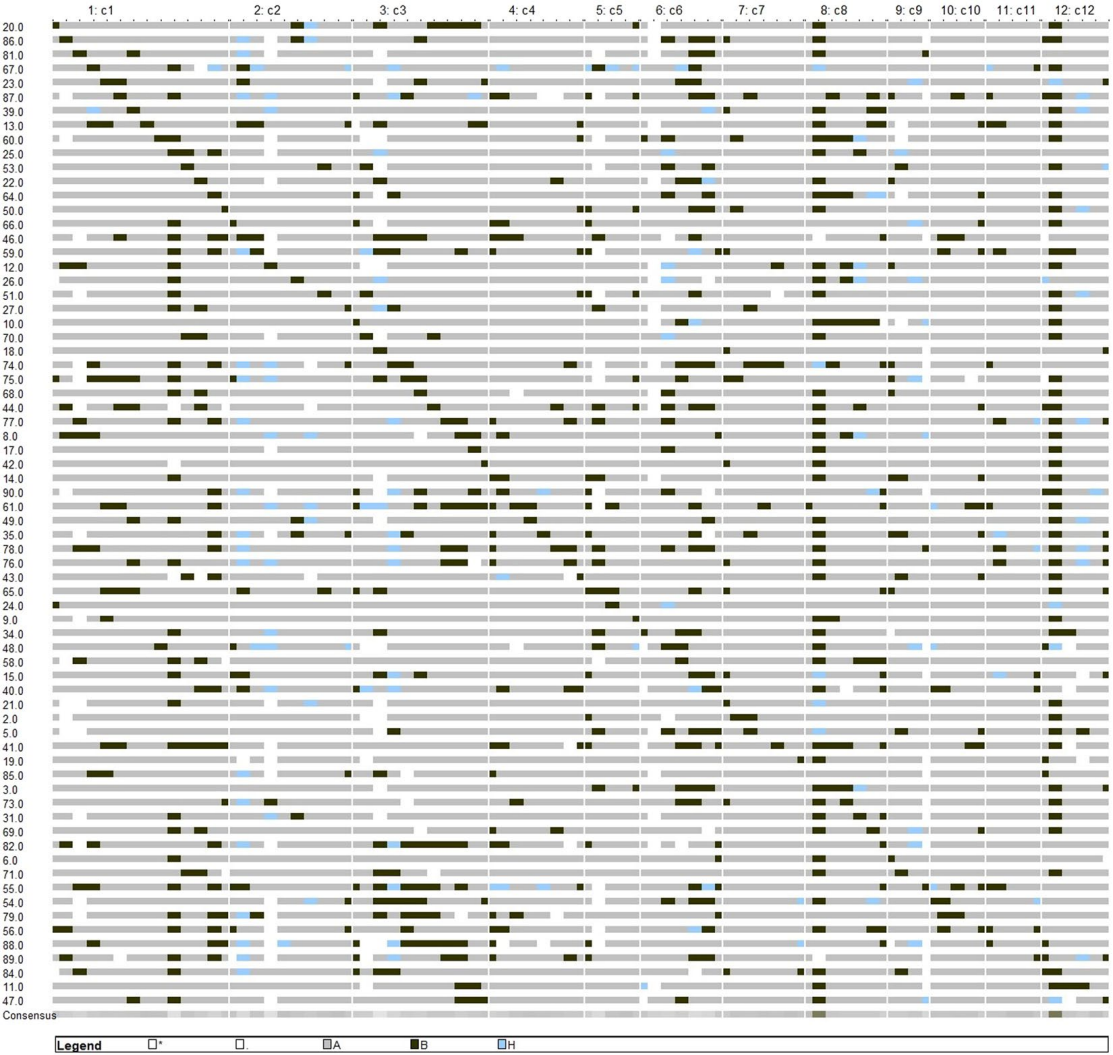
Graphical genotypes of 70 Swarna/*O. nivara* chromosomal segment substitution lines using 140 SSRs in BC_2_F_8_ (grey - Swarna homozygous, black - *O. nivara* homozygous, blue -heterozygous, white - missing data).

When genotypic data was subjected to CSSL finder, 70 out of 90 lines formed a minimal set of CSSLs with 11.94% of donor and 88.6% of recurrent parent segments (Fig. 1). The minimum donor genome in a CSSL was 2.33% in NK24 and maximum was 26.51% in NK61. NK61 was significantly better than the Swarna for YLDP and PDP and negatively significant for DFF and DM. NK24 was not significant for any trait. The number of substituted chromosome segments per line ranged from 2 to 16 with an average of 8 in the CSSL set and the number of segments observed per chromosome were 2 to 11 with an average of 6 segments. 70 CSSLs represented 94.4% of *O. nivara* genome but the donor genome was absent in small regions on chromosomes 5, 6, 9, 11 and 12. Chromosomes 5, 9, 11 and 12 were represented by 3 CSSLs each and chromosome 1 by 14 CSSLs. The line NK24 had two donor segments on chromosomes 1 and 5 while CSSLs NK2, NK11, NK18, NK19 and NK21 had only three segments each and on different chromosomes (Supplementary Table 7). NK22 with 91.86% Swarna background and 8.14% *O. nivara* introgression of 120.31 cM showed only six substituted segments. Similarly, NK50 had the lowest size of 5.82 cM *O. nivara* segment substitutions with 84.88% Swarna alleles and 15.12% of donor alleles.

### Construction of linkage map and QTL mapping

**Figure 2:**
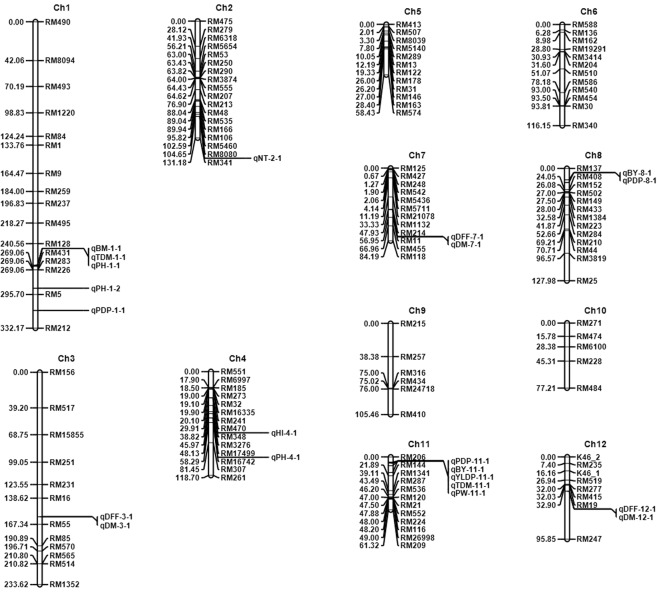
Molecular linkage map of 140 SSRs with position of QTLs for 12 agronomic traits mapped using ICIM detected from the mean phenotypic data of 3 years.

QTL detection was carried out using composite interval mapping (CIM), mapping of additive and digenic epistasis genes with chromosome segment substitution lines (CSL) and QTL-by-environment interaction analysis (MET) to dissect the genetic basis of yield traits. Consistent detection of QTLs was observed across the analytical methods in same genomic region. Eight chromosomes (chromosome 1, 2, 3, 4, 7, 8, 11 and12) showed significant genomic regions associated with major QTLs for yield traits (Fig. 2). LOD scores of QTLs ranged from 2.55 to 22.98 and explained PVE from 6.51% to 46.77% (Table 3). QTLs were detected above threshold LOD values for all the traits except for number of productive tillers after 1000 permutations. 3 QTLs each for days to flowering and days to maturity were detected in chromosome 3, 7 and 12. For plant height 3 QTLs *qPH1.1, qPH1.2* and *qPH4.1* were mapped. One QTL each was identified for number of tillers (*qNT2.1*), panicle weight (*qPW11.1*), grain yield (*qGY11*.1), biomass (*qBM1.1*) and harvest index (*qHI4.1*). Two QTLs, *qBY8.1* and *qBY11.1* were mapped for bulk yield and *qTDM1.1* and *qTDM11.1* for total dry matter. For per day productivity (PDP) three QTLs were detected in chromosomes 1, 8 and 11. The QTL on chromosome 1 with the flanking markers RM128 and RM431 was associated with PH, BM and TDM and explained phenotypic variation of 46.77%, 27.11% and 15.17% respectively and the trait- increasing alleles were from *O. nivara*. This was also confirmed by single marker analysis and interval mapping (Supplementary Table 8 and 9). The QTL on chromosome 11 in the region between RM206 and RM144 showed significant effects for grain yield with PVE of 18.92%. The same region harboured QTLs for PW with 15.40%, BY with 15.91% and PDP with 15.46% phenotypic variation. The QTL flanked by RM137 and RM408 on chromosome 8 was associated with PDP and BY. This QTL explained 9.58% of the variation for PDP and 10.85% for BY. A QTL was identified in chromosome 2 with PVE of 14.19% for NT and in chromosome 4 with a PVE of 14.88% for HI. DFF and DM showed common QTL between RM16 and RM55 in chromosome 3, between RM214 and RM11 in chromosome 7 and between RM19 and RM247 in chromosome 12 with PVE ranging from 16.15% to 26.33%. Additive effect showed that trait enhancing alleles for PH, BM, PW, GY, TDM and PDP were contributed from the donor parent *O.nivara* while the alleles for DFF and DM were from Swarna.

**Table 3:** Additive effect QTLs for yield traits based on phenotyping under three environments E1, E2, E3 and detected using QTL IciMapping 3.2.

QTL	Trait	Chr	Pos	Left Marker	Right Marker	LOD	PVE(%)	Add	LOD			PVE(%)			Additive effect		
									E1	E2	E3	E1	E2	E3	E1	E2	E3
*qBM1.1*	BM	1	264	RM128	RM431	5.95	27.11	−6.11	3.55	5.17	8.49	15.09	22.73	33.26	−3.94	−6.35	−6.84
*qPH1.1*	PH	1	265	RM128	RM431	22.98	46.78	−23.38	18.90	18.15	24.46	44.11	49.47	38.74	−24.92	−21.03	−26.14
*qTDM1.1*	TDM	1	264	RM128	RM431	3.19	15.18	−7.07	_	3.20	4.70	_	15.96	19.99	_	−8.13	−7.86
*qPH1.2*	PH	1	289	RM226	RM5	3.34	6.59	−7.44	4.89	_	_	11.96	_	_	−11.64	_	_
*qPDP1.1*	PDP	1	313	RM5	RM212	2.55	11.69	−0.02	_	_	_	_	_	_	_	_	_
*qNT2.1*	NT	2	107	RM8080	RM341	4.24	14.19	−2.68	3.18	3.69	5.03	15.32	13.07	11.28	−2.73	−2.45	−2.34
*qDFF3.1*	DFF	3	159	RM16	RM55	6.42	18.28	5.17	7.55	4.55	6.24	16.60	16.16	18.20	4.43	5.86	5.17
*qDM3.1*	DM	3	159	RM16	RM55	5.75	17.50	5.02	5.59	4.51	5.75	15.00	16.07	17.50	4.13	5.84	5.02
*qPH4.1*	PH	4	53	RM17499	RM16742	3.71	6.51	18.43	_	_	5.01	_	_	7.29	_	_	21.87
*qHI4.1*	HI	4	30	RM470	RM348	2.78	14.88	−5.18	_	_	_	_	_	_	_	_	_
*qDFF7.1*	DFF	7	48	RM214	RM11	7.50	16.35	9.54	8.50	6.07	7.40	15.82	14.23	16.32	8.38	10.80	9.55
*qDM7.1*	DM	7	48	RM214	RM11	7.21	16.16	9.43	7.46	6.05	7.21	15.33	14.21	16.16	8.22	10.77	9.43
*qBY8.1*	BY	8	13	RM137	RM408	2.79	10.85	−100.29	_	5.35	_	_	22.89	_	_	−176.37	_
*qPDP8.1*	PDP	8	16	RM137	RM408	3.63	9.58	−0.03	3.90	3.83	_	11.46	16.04	_	−0.05	−0.02	_
*qBY11.1*	BY	11	7	RM206	RM144	4.80	15.92	−103.27	_	_	5.51	_	_	17.43	_	_	−125.93
*qPDP11.1*	PDP	11	6	RM206	RM144	5.73	15.47	−0.03	_	4.53	4.23		22.09	17.63	_	−0.03	−0.03
*qPW11.1*	PW	11	18	RM206	RM144	2.68	15.41	−0.32	_	_	_	_	_	_	_	_	_
*qTDM11.1*	TDM	11	10	RM206	RM144	4.46	15.94	−8.79	3.01	4.17	3.63	10.17	12.26	14.16	−10.23	−9.24	−8.04
*qGY11.1*	GY	11	7	RM206	RM144	5.17	18.93	−4.05	_	4.51	4.37	_	23.30	15.98	_	−4.13	−4.23
*qDFF12.1*	DFF	12	40	RM19	RM247	10.16	26.30	5.85	12.72	6.68	9.90	28.76	20.53	26.28	5.57	6.16	5.85
*qDM12.1*	DM	12	40	RM19	RM247	9.65	26.33	5.80	10.69	6.66	9.66	27.77	20.53	26.33	5.11	6.15	5.80

LOD threshold values at10000 permutations: DFF-8.9495, DM-6.0825, PH-9.5269, NT-3.2942, NPT-2.3922,PW-1.6816,GY-5.0034,BM-2.4836,BY-3.767,TDM-2.8184,HI-1.8661,PDP-3.8528. DFF- days to 50% flowering, DM- days to maturity, PH- plant height, NT- number of tillers, NPT- number of productive tillers, PW- panicle weight, GY- yield per plant, BM- Biomass, BY- bulk yield, TDM-total dry matter, HI- harvest index, PDP- per  day productivity. Chr-chromosome No. Pos-Position cM, LOD -Logarithm of odds ratio, PVE - Phenotypic variance explained, E1-wet season 2014, E2-wet season 2015, E3-wet season 2016.

### MET QTL mapping

Three years phenotypic data was subjected to MET functionality to detect QTL by environment interaction in 90 BILs. 31 significant QTLs were detected above threshold LOD MET under 1000 permutations with a probability value (PIN) of 0.0001. METQTLs were identified for the traits on all chromosomes except 6 and 9. The PVE by a single QTL ranged from 3.17% to 45.88% at LOD levels from 4.11 to 58.91 (Table 4). QTLs for DFF and DM at chromosome 7 (RM214-RM11) and chromosome 12 (RM19- RM247), PW at chromosome 11 (RM206-RM144) and PH at chromosome 1 (RM128-RM431) were found to have high LOD (AxE). PW and BY QTL at chromosome 11 (RM206-RM144) and BY QTL at chromosome 8 (RM137- RM408) showed highest QTL by environment phenotypic variance. A significant QTL for panicle weight between RM206 and RM144 in chromosome 11 with a PVE of 16.50% was identified and showed highest additive by environment PVE of 3.41%. Two QTLs on chromosomes 8 and 11 for bulk yield were detected with large additive effects across 3 environments and 14.07% and 9.67% PVE respectively.

**Table 4:** QTLs identified through MET analysis for yield traits based on phenotyping under three environments E1, E2, E3 and detected using QTL IciMapping 3.2.

Trait	Chr	Pos	Left Marker	Right Marker	LOD	LOD(A)	LOD (A×E)	PVE	PVE(A)	PVE (A×E)	Add	A×E1	A×E2	A×E3
BM	1	264	RM128	RM431	16.56	16.07	0.49	24.39	24.3	0.09	−5.62	0.43	−0.01	−0.42
HI	1	269	RM128	RM431	4.44	4.38	0.05	5.26	4.62	0.64	2.4	1.24	−0.84	−0.4
PH	1	264	RM128	RM431	58.91	57.58	1.33	45.88	45.38	0.49	−23.14	−1.25	3.37	−2.12
TDM	1	266	RM128	RM431	8.93	8.34	0.59	12.4	12.24	0.17	−5.54	0.86	−0.18	−0.68
GY	1	264	RM128	RM431	16.56	16.07	0.49	24.39	24.3	0.09	−5.62	0.43	−0.01	−0.42
PH	1	292	RM226	RM5	6.77	6.28	0.49	3.92	3.26	0.66	−5.28	−3.02	2.77	0.25
NPT	2	109	RM8080	RM341	5.94	5.93	0.01	9.65	9.65	0	−1.73	−0.02	0.05	−0.04
NT	2	108	RM8080	RM341	10.8	10.76	0.04	12.38	12.37	0.02	−2.62	−0.03	0.13	−0.1
DFF	3	160	RM16	RM55	18.26	17.2	1.06	13.46	13.29	0.18	5.05	−0.71	0.71	−0.01
DM	3	160	RM16	RM55	15.73	15.11	0.62	15.96	15.71	0.25	4.79	−0.73	0.75	−0.02
HI	3	131	RM231	RM16	4.11	4.01	0.09	4.37	4.35	0.03	−2.5	−0.25	0.04	0.22
DFF	3	172	RM55	RM85	14.87	13.99	0.88	12.03	11.86	0.16	5.04	−0.71	0.73	−0.02
HI	4	30	RM470	RM348	4.13	3.9	0.24	4.04	3.92	0.12	−4.01	−0.55	−0.45	0.99
PH	4	53	RM17499	RM16742	8.81	8.6	0.21	5.56	5.51	0.05	19.19	−0.39	−1.99	2.37
HI	5	0	RM13	RM507	4.64	4.35	0.29	4.38	4.38	0.01	−2.23	0.13	−0.07	−0.06
DFF	7	48	RM214	RM11	21.97	19.06	2.91	12.97	12.83	0.14	9.58	−1.19	1.22	−0.03
DM	7	48	RM214	RM11	20.72	18.81	1.91	16.3	16.1	0.2	9.47	−1.26	1.3	−0.04
BY	8	20	RM137	RM408	6.3	5.82	0.48	9.67	8.9	0.78	−88.44	17.6	−36.95	19.35
PDP	8	18	RM137	RM408	7.64	7.48	0.16	14.55	14.02	0.53	−0.03	−0.01	0	0
TDM	8	19	RM137	RM408	4.69	4.67	0.02	6.69	6.63	0.06	−8.61	−0.94	−0.05	0.99
HI	10	30	RM6100	RM228	4.56	3.94	0.63	4.88	4.47	0.41	−4.12	0.75	1.02	−1.76
BM	11	13	RM206	RM144	6.17	6.15	0.02	8.48	8.36	0.13	−4.15	−0.61	−0.02	0.63
BY	11	3	RM206	RM144	7.58	7.15	0.42	14.07	13.13	0.94	−62.75	18.21	4.06	−22.27
PDP	11	3	RM206	RM144	9.54	8.74	0.8	14.96	14.65	0.31	−0.02	0	0	0
PH	11	19	RM206	RM144	5.88	5.23	0.65	3.17	2.87	0.3	−7.8	1.41	2.14	−3.54
PW	11	21	RM206	RM144	6.53	4.7	1.82	16.5	13.08	3.41	−0.26	−0.17	0.03	0.14
TDM	11	10	RM206	RM144	10.37	10.2	0.17	15.35	15.29	0.06	−8.1	−0.48	−0.24	0.72
GY	11	13	RM206	RM144	6.17	6.15	0.02	8.48	8.36	0.13	−4.15	−0.61	−0.02	0.63
NPT	11	48	RM224	RM116	4.66	4.57	0.09	5.09	4.79	0.3	0.53	0.19	−0.1	−0.08
DFF	12	40	RM19	RM247	29.21	27.04	2.17	19.84	19.78	0.07	5.73	−0.39	0.41	−0.02
DM	12	39	RM19	RM247	26.88	24.93	1.95	24.86	24.79	0.07	5.58	−0.34	0.38	−0.04

DFF- days to 50% flowering, DM- days to maturity, PH- plant height, NT- number of tillers, NPT- number of productive tillers, PW- panicle weight, GY- yield per plant, BM- Biomass, BY- bulk yield, TDM-total dry matter, HI- harvest index, PDP- per  day productivity, Chr- Chromosome, Pos- Position in cM, LOD -Logarithm of odds ratio, PVE - Phenotypic variance explained, ADD- Additive effect, E1-wet season 2014, E2-wet season 2015, E3-wet season 2016.

Among the QTLs identified from 90 BILs data through ICIM, 10 were detected across 3 environments for DFF, DM, PH, NT or TDM consistently and 4 were detected in at least 2 environments for PDP and GY and with a PVE above 10% (Supplementary Table 10). QTLs that were mapped to the genomic regions located within the same set of flanking markers were considered as representing the same QTL. The QTL region within RM128 and RM431 on chromosome 1 showed PVE ranging from 38.74 to 44.11% for PH across the environments, and the same region showed QTL for BM also in all the 3 years. Similarly, the locus between RM16 to RM55 at chromosome 3, RM214 to RM11 at chromosome 7 and RM19 to RM247 at chromosome 12 showed major QTLs for DFF and DM consistently over the years. QTL for NT was detected between RM8080 and RM341 at chromosome 2 in all the 3 years, with PVE varying between 11.28 to 15.32%. Similarly, region RM206 to RM144 at chromosome 11 was consistently identified for TDM. QTLs for GY and PDP were detected on chromosome 8 (RM137-RM408) and chromosome 11 (RM206-RM144), in 2 consecutive years.

The locus RM128-RM431 at chromosome 1 showed QTLs for grain yield, harvest index, biomass, total dry matter and plant height by multi environment testing (MET) QTL mapping and also with high PVE and LOD. Another region RM137-RM408 at chromosome 8 had QTLs for total dry matter, bulk yield and per day productivity and QTLs showed additive by environment interaction. Similarly, the region between RM206 and RM144 on chromosome 11 also harboured QTLs for plant height, biomass, total dry matter, bulk yield, grain yield, per day productivity and panicle weight showing high PVE, PVE (additive) and PVE (additive by environment). 21 out of 31 QTLs showed negative additive effects, indicating that the majority of introgressed segments from *O.nivara* in these regions contributed to trait enhancing alleles.

### QTL mapping using CSSL set

A likelihood ratio test based on stepwise regression-based likelihood ratio test (RSTEP-LRT) was used to map QTLs in marker defined chromosomal regions of 70 non-idealized CSS lines. This method ensures accuracy of QTL detection using CSSLs. The results of QTL mapping from BILs and CSSL set or a subset representing the whole donor genome of mapping population was compared.

Altogether 10 marker loci were identified as significantly linked with trait improvement using 3 environment data (Table 5). Among these RM214, RM55 for DFF and DM; and RM431 for PH were consistently associated with trait increase in three environments and also the average environment. RM206 showed trait increase for BY, PDP and GY in 2015, 2016 and in mean. Similarly RM25 was a significant locus for panicle weight in CSL QTL mapping for two environments. These markers also showed significant trait linkage when analysed through single marker analysis using the complete set of back cross introgression lines. The mean value of the QTL genotype of parents M(QQ) and M(qq) showed that Swarna has higher values of QTL genotype for all traits except DFF and DM.

**Table 5:** QTLs identified through CSL analysis for yield traits based on phenotyping under three environments E1, E2, E3 and detected using QTL IciMapping 3.2.

Trait	Chr	Marker	LOD				PVE				Additive Effect			
			E1	E2	E3	Mean	E1	E2	E3	Mean	E1	E2	E3	Mean
BM	1	RM431	_	_	2.95	_	_	_	16.70		_	_	−3.88	_
PH	1	RM431	6.40	6.25	7.58	7.41	28.20	31.64	34.47	36.11	−16.57	−13.56	−16.29	−15.70
PW	2	RM279	3.84	_	_	_	22.40	_	_	_	−0.29	_	_	_
DFF	3	RM231	4.31	_	_	_	16.97	_	_	_	3.19	_	_	_
DM	3	RM231	4.16	_	_	_	17.06	_	_	_	3.28	_	_	_
DFF	3	RM55	4.07	4.70	5.62	5.68	15.64	19.23	21.75	21.85	3.73	6.00	5.59	5.56
DM	3	RM55	3.55	4.68	5.49	5.49	14.19	19.16	21.28	21.29	3.64	5.98	5.51	5.51
PH	4	RM307	_	_	2.88	_	_	_	11.45	_	_	_	−11.65	_
DFF	7	RM214	6.06	5.72	6.42	6.50	24.83	23.87	25.36	25.58	8.53	12.11	10.94	10.91
DM	7	RM214	5.36	5.70	6.29	6.29	22.85	23.82	25.03	25.02	8.38	12.08	10.82	10.82
PW	8	RM25	_	2.83	2.54	2.97		17.61	15.93	18.28	_	−0.23	−0.25	−0.23
PDP	8	RM408	4.15	_	_	_	20.18	_	_	_	−0.07	_	_	_
GY	8	RM408	3.23	_	_	_	19.16	_	_	_	−8.67	_	_	_
BY	11	RM206	_	3.37	3.60	3.80	_	19.89	21.11	22.10	_	−74.32	−83.53	−74.63
HI	11	RM206	_	2.53	2.91	2.81	_	15.36	17.54	17.93	_	−3.27	−3.83	−3.13
PDP	11	RM206	_	4.40	3.68	4.31	_	25.13	21.48	24.69	_	−0.02	−0.02	−0.02
GY	11	RM206	_	3.77	3.30	3.66	_	21.96	19.53	21.42	_	−3.01	−2.93	−2.85
PH	12	RM235	3.36	_	_	_	13.25	_	_	_	8.90	_	_	_

DFF- days to 50% flowering, DM- days to maturity, PH- plant height, NT- number of tillers, NPT- number of productive tillers, PW- panicle weight, GY- yield per plant, BM- Biomass, BY- bulk yield, TDM-total dry matter, HI- harvest index, PDP- per  day productivity, Chr- Chromosome, Pos- Position in cM. E1-wet season 2014, E2-wet season 2015, E3-wet season 2016, LOD -Logarithm of odds ratio, PVE - Phenotypic variance explained

### Epistatic QTL

**Figure 3:**
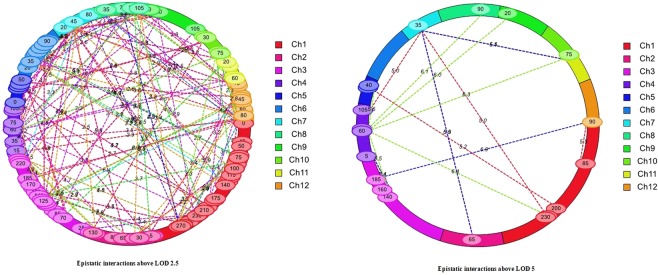
Epistatic QTLs identified in Swarna/*O. nivara* back cross introgression lines using 140 SSRs in BC_2_F_8_ (Epistatic interactions above LOD 2.5, Epistatic interactions above LOD 5).

17 significant epistatic QTL were detected from 90 BILs for DFF, DM and PH at threshold LOD > 5 with PVE ranging from 2.44 to 25.85% (Table 6). Other traits did not show any epistasis above LOD 5. At LOD > 3 we found minor interactions between number of loci across chromosomes (Fig. 3). A total of 6 digenic interactions were detected for days to fifty percent flowering. Interestingly, three of them occurred between significant main effect QTLs for DFF and non-significant loci in other chromosomes. A negative digenic interaction involving loci at chromosome 1 and chromosome 5 was detected and both additive effects were from *O. nivara*. Four of the epistatic interactions for DM were similar in case of DFF involving the main effect QTLs identified through ICIM. However, two different loci RM493-RM1220 on chromosome 1 and RM55-RM85 on chromosome 3 showed epistatic interaction with the same locus RM19-RM247 on chromosome 12 for DFF and DM respectively. An epistatic interaction between chromosome 3 (RM55-RM85) and chromosome 12 (RM19-RM247) explained a high phenotypic variance of 25.85%. Six digenic loci showing epistatic interactions were detected for plant height. Single locus RM16742-RM307 on chromosome 4 showed interactions with loci in chromosomes 1, 3, 5, 8, 9 and 10. It is worthwhile to note that the epistatic interactions had slightly decreased LOD values of already detected main effect QTLs. The total PVE explained by the epistatic QTLs was lower than the PVE of main effect QTLs for plant height.

**Table 6:** Epistatic QTLs influencing yield traits based on adjusted mean values from three environments E1, E2, E3.

Trait	Chr1	Pos1	Marker interval 1		Chr 2	Pos2	Marker interval 2		LOD	PVE (%)	Add1	Add2	Add ×Add
DFF	3	140	RM16	RM55	4	5	RM551	RM6997	5.44	8.86	2.70	2.25	−3.97
DFF	1	200	RM237	RM495	5	35	RM163	RM574	5.19	10.08	−1.55	−2.57	4.85
DFF	1	225	RM495	RM128	7	35	RM1132	RM214	5.02	10.51	5.85	6.08	−7.32
DFF	2	65	RM207	RM213	7	35	RM1132	RM214	5.93	9.89	5.01	4.51	−5.54
DFF	4	105	RM307	RM261	7	35	RM1132	RM214	5.03	9.67	5.82	6.33	−7.47
DFF	7	35	RM1132	RM214	11	0	RM206	RM144	5.50	10.47	5.51	3.93	−5.73
DFF	1	85	RM493	RM1220	12	90	RM19	RM247	5.53	14.28	3.91	−2.82	2.27
DM	3	140	RM16	RM55	4	5	RM551	RM6997	5.11	9.70	2.59	2.26	−3.90
DM	2	65	RM207	RM213	7	35	RM1132	RM214	5.54	10.96	5.03	4.49	−5.45
DM	7	35	RM1132	RM214	11	0	RM206	RM144	5.11	11.74	5.47	3.87	−5.59
DM	3	185	RM55	RM85	12	90	RM19	RM247	6.92	25.85	2.54	−4.32	4.47
PH	1	230	RM495	RM128	4	60	RM16742	RM307	6.39	4.19	12.35	4.24	−17.96
PH	3	160	RM16	RM55	4	60	RM16742	RM307	5.54	2.72	14.88	3.16	−15.73
PH	4	60	RM16742	RM307	5	40	RM163	RM574	5.59	2.72	2.28	16.12	−14.39
PH	4	65	RM16742	RM307	8	90	RM44	RM3819	6.05	3.38	5.41	12.14	−17.83
PH	4	60	RM16742	RM307	9	20	RM215	RM257	6.00	4.39	6.13	10.74	−19.26
PH	4	60	RM16742	RM307	10	75	RM228	RM484	6.34	2.44	3.37	14.61	−15.49

DFF- days to 50% flowering, DM- days to maturity, PH- plant height, Chr- Chromosome, Pos- Position in cM. LOD -Logarithm of odds ratio, PVE - Phenotypic variance explained, ADD- Additive effect, E1-wet season 2014, E2-wet season 2015, E3-wet season 2016.

### Ethics statement

The authors declare that the experiments comply with the current laws of the country in which they were performed and in compliance with ethical standards.

## Discussion

Swarna is one of the most popular mega varieties in India and other rice growing countries and well-known for its high yield, long duration and stable performance across different ecosystems^33^. Introgressions from wild species *O.nivara* are reported to improve yield traits in Swarna and a variety DRRDhan 40 has also been released^34^. Improved backcross introgression lines with favourable agronomic traits and high yield stability were also identified through multi environment trials in Swarna background^29^. In this study the advanced back cross introgression lines (BC_2_F_8_) of Swarna/*O.nivara* were phenotyped for three years for yield and related traits and transgressive segregation with significantly positive and negative difference with the recurrent parent was observed (Supplementary Fig. 2) indicating the existence of allelic variations for each trait in each parent. Continuous frequency distribution was observed for all traits demonstrating quantitative inheritance of the traits. DFF, DM, NT, NPT, GY and PDP showed kurtosis values above 1 and among these only PDP showed +ve skewness. Except these, the absolute values of skewness and kurtosis were less than 1 for all traits, and showed normal distribution indicating the suitability for QTL analysis. In addition, PH, BM, BY and TDM also showed higher standard deviations suggesting significant effect of environment in their phenotypic expression.

Significant pair wise comparison showed 31 lines significantly different than Swarna for atleast one trait and 27 lines showed significant difference for more than one trait. This suggests interspecific combination of two divergent parents results in accumulation of superior alleles with additive and complementary effects^35^ which can help to not only broaden the genetic base but create desirable novel variation in cultivar gene pools. Lines NK58 and NK83 showed significantly higher bulk yield, biomass and plant height and are valuable breeding materials for yield improvement. NK61 which showed significantly higher grain yield than Swarna and significantly lower days to flowering and maturity has commercial value as an early, high yielding breeding line. A set of lines which are either positively or negatively significant over Swarna for days to flowering and days to maturity were identified in this biparental population and they are useful in dissecting the genetics of heading date in rice.

### Yield QTL, ICIM, MET, EPI

Among the 22 major QTLs identified in this study through ICIM, all the QTLs except those for DFF, DM and one QTL for PH showed trait enhancing alleles from *O. nivara*. This indicates that in general introgression of alleles from *O. nivara* into the elite cultivar background had a beneficial effect on improving most of the yield traits. Major QTL for PH at region flanked by RM128 and RM431 on chromosome 1 was the most consistent across all 3 environments with increasing effect from *O. nivara* and explained the highest proportion of PVE (15–33%) and both the flanking markers were previously reported to be associated with plant height^20,21,32,36^. This establishes the robustness of mapping QTLs in our study. Another QTL for plant height was found between RM226 and RM5 in chromosome 1. Kadambari *et al*.^37^ reported a major QTL alternate semi-dwarf 1 (*asd1*) governing plant height on chromosome 1(chr01:2559897–2564321) approximately 36 Mb upstream from *sd1*. Several factors, such as the number of detected QTL, the degree of linkage of markers with the genes, the range of variation within the population, the population size, genetic background and phenotyping environments influence the magnitude of phenotypic variation explained by a QTL^38^. The main limitation in using QTLs for marker assisted selection and breeding is the poor consistency of QTL effects across environments and so stable QTLs need to be identified^39,40^. Dissection of candidate chromosomal regions to elucidate the genetic mechanisms for grain yield, using multi-environment testing is a key approach in crop improvement. Therefore, to detect such chromosomal regions we conducted MET QTL mapping by validating the phenotypic performance in three environments for accurate mapping and effective use of QTL/genes.

QTLs for DFF and DM colocalised in three regions on chromosomes 3, 7 and 12 and explained more than 55% cumulative phenotypic variance in these regions for each trait. Thomson *et al*.^41^ identified a stable QTL *dth7.1* with peak marker RM214 using BC_2_F_2_ population *O. sativa* cv. Jefferson/ *O.rufipogon* IRGC105491 in the same chromosome region as in our study. RM11, the other flanking marker of *qDFF7.1* and *Qdm7.1*, was also reported as linked to heading date^42,43^. The region flanked by RM214 - RM11 on chromosome 7 was reported as a major QTL *Qhd7*^42^, *Hd4*^44^, *dth7.1*^41,42^, *qDTH-7–1*^45^ and *hd7*^46^ for heading date. Similarly, flanking markers (RM16-RM55) for *qDFF3.1* were associated with QTLs *dth3.2, dth3.3, dth3.4*^41,47^ and RM19 of *dth12.1* was reported linked to heading date previously^48^. Interestingly these regions were also reported as linked to several other traits e.g. yield per plant, grain width, number of tillers per plant, spikelet fertility and grain weight^18,36,49,50^ (Supplementary Table 11). QTL analysis for heading date conducted over five years located stably expressing QTL on chromosomes 3^51^.

The QTL *qPW11.1* flanked by markers RM206 and RM144 was detected for PW on chromosome 11 and explained 15.40% of the phenotypic variation. The regions RM128-RM431 at chromosome 1, RM137-RM408 at chromosome 8 and RM206-RM144 at chromosome 11 showed clusters of significant QTLs for various yield traits with QTL by environment interaction. The chromosome 11 cluster had QTLs for panicle weight, grain yield, bulk yield, total dry matter and per day productivity. RM206 was reported to be significantly associated with panicle length, grain sterility, spikelet setting density, percent sterility, plant height, number of productive tillers, yield per plant, 1000 grain weight, panicle diameter and seed shattering^18,52,53^. Marathi *et al*.^52^ reported that RM144 was linked to panicle length and yield per plant. A major QTL containing genes *Pikh*/*Pi54* was previously detected between RM206 and RM144^54^ for resistance to neck blast and leaf blast^55,56^. RM206 is also reported as linked to canopy temperature at reproductive stage in drought^57^. RM408 was found closely linked to multi-tiller and dwarf mutation on the short arm of chromosome 8^58^.

These traits were also correlated and have contributory effect on each other. Wang *et al*.^38^ suggested pleiotropic effects of individual locus or tight linkage between loci in the same region is responsible for significant correlation between traits. The nature and direction of these strong associations suggests that these traits are under similar genetic control, conferred by pleiotropic effects of the underlying genes. It is mainly due to cluster of co-expressed genes serving different functions and selection of these QTLs may help in simultaneous improvement of these traits^59^. The QTL effect always depends on the background genome as well as direction of correlation among the traits even though the QTLs for correlated traits are colocalised^60^. Development and phenotyping of segregating population of recombinants of these regions are promising in unravelling genetic mechanisms underlying these regions^61^.

Grain yield and yield component traits are typically quantitative in nature and are influenced by environment and epistatic interactions^62^. A total of 10 putative major effect QTLs were detected across three environments and 31 QTLs with significant LOD values were identified for all yield traits. QTL region flanked by RM206 and RM144 at chromosome 11 for panicle weight was detected with significant LOD values and highest PVE additive **×** environment interaction suggesting it as a stable QTL. Even though, QTLs related to days to flowering, maturity and plant height were consistent with significant LOD levels their phenotypic variance in MET analysis was low indicating major effect of environment and the environment interaction on phenotypic expression of these traits. Hosseini *et al*.^63^ detected QTL clusters for rice grain appearance quality traits with main, epistatic and QTL×environment interaction effects using two populations of backcross inbred lines (BILs) and suggested that co-located QTL or genes with pleiotropic effects could control these traits. Li *et al*.^48^ carried out QTL **×** E interaction studies of rice doubled-haploid lines in nine diverse environments for heading date and plant height and found varying effects of QTL both in magnitude and direction due to more pronounced epistatic effects and non expression of some QTLs, weak expression or their different expression in different environments. Quantitative traits are also influenced by interaction between non allelic genes and QTL × environment interactions^63^ and even the minor effect interactions influence trait expression significantly. It is observed that in most of the cases, complete effects of detected QTLs are not expressed in advanced generations or in marker assisted selection. This is mainly due to the fact that generally the major effect of QTLs are determined only by the additive component, however epistatic and environmental interactions are mostly ignored. Simultaneous detection of major effect, epistatic and environmental interaction is required for unbiased determination of QTL effect and breeding value^64,65^.

A total of 17 pair wise epistatic interaction QTLs were in this study above a threshold LOD of 5 with significant additive × additive (AA) effects and showed interaction loci for either days to flowering or plant height. We found only 17 significant epistatic interactions but a large network of weak epistatic interactions among yield traits. Additive by additive effects of epistatic QTLs were comparatively lower than additive effect of any of the corresponding QTL. This might be mainly due to presence of few individuals with rare loci combinations in the populations, interference from other loci and low epistatic variance from interactions^66^.

Interestingly, all the pair wise interacting loci for days to flowering and maturity involved at least one of the major QTL except an interaction QTL pair on chromosome 1 and 5. 7 pairs of loci showed epistatic interaction for days to 50% flowering (DFF) and 3 of them also showed interaction effects for days to maturity (DM) and the fourth pair of epistatic loci for DM had only one common QTL with DFF^48,67,68^. Yang *et al*.^69^ characterized epistatic interaction between heading date genes of different chromosomes in rice. Exploration of epistatic interactions among multiple QTLs improved the understanding on genetic network of flowering and the information is useful for pyramiding of QTLs. Subudhi *et al*.^70^ demonstrated the presence of epistatic interactions and found RM214 linked to Ghd7/ *qHD7–1* is involved and, in our study, also RM214 was one of 5 pairs of epistatic interacting loci among different flowering related QTLs. Similar to our study, Nemato *et al*.^71^ and Zhang *et al*.^72^ also reported the involvement of novel epistatic loci (which are not detected in composite interval mapping) in regulation of flowering. Yadav *et al*.^73^ reported the existence of epistatic interactions between QTLs that influence the expression of a complex trait such as grain yield under stress and non-stress conditions.

The QTL for plant height at chromosome 4 (RM16742-RM307) showed interactions with all the 6 loci for plant height on other chromosomes. However, these 6 interacting epistatic loci for PH did not involve the major QTL for plant height close to semidwarf gene at chromosome 1 suggesting its independent major effect. Zhao *et al*.^74^; Shang *et al*.^75^ reported that digenic epistasis was generally not observed among detected major QTLs; as observed in our study. Thomson *et al*.^41^ showed that qDTH-4–3(t) (dth4.1) located on chromosome 4 and closely linked with marker RM307 controls days to heading. Zhao *et al*.^76^ mapped a semidwarf gene *sd-t2* closely linked to RM307. Gaikwad *et al*.^19^ also reported that *O. nivara* allele at RM307 was responsible for increased plant height in introgression lines. In our study this QTL even with a small additive effect from *O. nivara* showed a complementary effect on other QTLs for PH indicating an important role of epistatic interaction in phenotypic variation. However, these epistatic loci showed significant interactions only in any two of three environments.

QTLs with main, epistatic and QTL and environment interaction effects were identified previously for salt tolerance^38^, yield traits^62^, grain chalkiness^74^ and heading date^77^ to dissect the genetic mechanisms of these complex traits. Zhang *et al*.^78^ reported ICIM is an efficient tool to find epistatic QTLs and unbiased by population type or size and the genetic effects from simulation studies using F_2_ and doubled haploid (DH) populations. Wang *et al*.^79^ reported that epistatic interaction effects depend on the significance and the direction of effects of participant QTL with a consistent trend towards negative epistatic effects as in the current study and these are of much importance when the QTLs/ gene involved are co introgressed or pyramided for crop improvement. Cumulative effects of QTL were significant for flowering duration and pyramiding QTL by marker-assisted selection would be an appropriate strategy to improve the traits showing cumulative or complementary epistasis^38^.

### **Yield QTL in BC**_**2**_**F**_**8**_**vs. BC**_**2**_**F**_**2**_

QTL mapping in this study was based on BILs and CSSLs at BC_2_F_8_ generation phenotyped for three years in normal irrigated conditions. The current mapping population was derived through single panicle selection from an earlier generation of BC_2_F_2_^16^. We compared the previous study where QTLs were detected with CIM and IM using QTL Cartographer in a population of 245 BC_2_F_2_ families and genotyped with 75 SSR markers; to understand the changes in QTL detection and QTL effect after 6 generations of selfing. Interestingly the single significant QTL for GY identified in the present study was also reported in the same region with a common flanking marker RM206 in the previous study. It is noteworthy, that QTL for plant height at chromosome 4, number of tillers at chromosome 2 and bulk yield at chromosome 8 were in the same region in both BC_2_F_2_ and BC_2_F_8_ generations showing a similar region of genetic control for components of biomass production. Population size and marker density directly affect the accuracy of QTL mapping^27,80,81^. However, in this study marker density was higher while population size was lesser than that of BC_2_F_2_ QTL mapping. The LOD levels for the same QTL were lower in BC_2_F_8_ than in BC_2_F_2_. A QTL for plant height showed highest LOD in both BC_2_F_2_ and BC_2_F_8_ with common flanking marker RM128. This region was previously reported for plant height and is close to semi dwarf gene *sd1*^20,32,82^ and QTLs for other yield contributing traits^21,83^.

Malathi *et al*.^20^ compared BILs of another mapping population Swarna/ *O.nivara* IRGC81842 and found only 3 out of 15 QTLs in BC_2_F_8_ were reported previously in BC_2_F_2_ generation. Similarly, Rangel *et al*.^10^ and Wickneswari *et al*.^84^ identified few common major QTLs across generations. Studies comparing previous QTL mapping with an advanced generation of RILs or back crosses found genetic distance of intervals was reduced resulting in more accurate mapping of QTLs with shorter average marker distances^75,85^. Since many major epistatic or environmental QTLs are context-dependent and do not appear across generations it is prudent to assess worthiness of a QTL for use in MAS. The advanced backcross generations also help in discovering minor QTLs with significant role in phenotypic expression due to a uniform genetic background which may have been masked in an early generation with large number of introgressions^86^. Any QTLs which are stable over generations, background and genomic context are high priority candidates for fine mapping, cloning, gene discovery and marker assisted breeding to improve the associated trait. Ten QTLs *qBM1.1, qDFF3.1, qDFF7.1, qDFF12.1, qDM3.1, qDM7.1, qDM12.1, qPH1.2, qNT2.1* and *qTDM11.1* are considered as stable QTLs in our study.

### CSSLs developed and identification of linked CSS

CSSL library was identified from this advanced back cross population (BC_2_F_8_) generated from an interspecific cross of *O. sativa* and *O. nivara* IRGC81832. Three sets of CSSLs were developed and QTLs were reported previously using different accessions of *O. nivara* by Malathi *et al*.^20^, Xin *et al*.^39^, Furuta *et al*.^87^, Ma *et al*.^88^. Subudhi *et al*.^70^ reported that the increased recurrent parent genome recovery of individual lines of CSSL helps in improved confidence of QTL by reducing the background noise in the mapping population. The presence of more than one chromosome segment substitution and missing segments is mainly because this population was generated after only two back crosses and no genomic or phenotypic selection during selfing. Incomplete genome coverage of wild accession is mainly attributed to inadvertent or deliberate selection in the early generation which may have removed the lines with donor segments contributing to any unfavourable trait in the population. Presence of genetic factors for cross incompatibility, hybrid sterility, lethality and recombination barriers which are well known in wild introgressions also are the probable reasons for missing segments in substitution lines. Chromosome segments in the CSSL genome composition graph showed uneven distribution of many segments on the two sides of the diagonal segments along with heterozygosity and missing segments and it might reduce mapping accuracy^89^. NK61 with maximum 26.51% alleles from *O. nivara* was the only CSSL showing significantly higher grain yield and lower flowering days compared to the parent Swarna. Thus, wild introgressions enhance yield and testing under large scale multilocation trials is required.

CSSLs with uniform genetic background harbouring specific chromosome segments from a donor species are ideal for mapping of QTLs^59,77^. Nine out of 20 QTL (45%) detected using CSSLs were observed in at least two environments and also based on mean data across environments. In comparison, only 14 out of 45 QTLs (31%) from the larger BIL population were detected in more than one environment indicating usefulness of CSSLs in identifying stable QTLs compared to primary mapping populations. It was demonstrated that CSSLs are more powerful than primary mapping populations like RIL and F_2_ to detect QTLs in multi environment testing using a set of inter specific CSSLs and RILs of cabbage (*Brassica rapa*)^79^. Most of the major QTLs were consistently detected across mapping populations and methods, however variation was observed in number, loci and phenotypic variance of many QTLs. In CSSLs the masking and interaction effects of multiple genes and loci are comparatively lower than in other mapping populations. This leads to more precise detection of observed variation in QTL mapping along with the genome effect of a background genotype^77,90,91^. Thus, CSSLs has the advantage of increasing the accuracy of QTL mapping while reducing the population size to be phenotyped, by representing the whole genome of the donor parent in a comparatively smaller subset.

Significant quantitative trait loci (QTLs) for different yield traits were located in 10 chromosomal segments above threshold levels and in at least one environment. These included QTLs for BM, BY, DFF, DM, HI, PDP, PH, PW and GY located in all chromosomes except 5, 6 and 9. The QTL locus near RM431 with large phenotypic variance for plant height and identified in 3 environments was previously identified as associated with *sd1* gene^20,21,32,82^. This may be a new allele for semi dwarf locus which can be confirmed by sequencing. RM431was reported as closely linked flanking marker for a major and consistent QTL *qDTY1.1* in multiple elite genetic backgrounds under both drought stress and non-stress condition^81^ and the same marker was used for transfer of the QTLs for yield under drought into different genetic backgrounds^92^.

The QTL locus near RM206 showed large phenotypic variance for BY, HI, PDP and GY with trait increase contributed by *O.nivara* alleles across two environments. QTL loci RM16-RM55 on chromosome 3 and RM214-RM11 on chromosome 7 for days to flowering and days to maturity showed low environment interaction and were consistently detected across the environments. But interaction between chromosome substitution and environment was not significant in case of regions at chromosome1 (RM431) for BM, at chromosome 2 for PW, at chromosome 3 for DFF and DM, at chromosome 4 and 12 for PH and at chromosome 8 for GY and PDP indicated by their low LOD (QTL**×**E). For enhancing grain yield, *O. nivara* allele at RM206 could be explored in breeding for wide adaptation. Wild donor genome segments detected through macro-segment mapping using CSSLs need further fine mapping to understand the genetic mechanisms for phenotypic expression^89^. Near isogenic lines and secondary mapping populations targeting these chromosome substitution segments are essential to assess the use of QTLs in MAS.

Identification of QTL candidate genes for yield and related traits would help in unravelling genotype to phenotype relationships. In genome wide QTL meta analysis reported by Swamy *et al*.^17^, PPR genes were associated with 15 out of 23 meta QTLs for yield. PPR genes are known to restore fertility in rice^40^. In the present study, cytochrome b561 underlies *qGY8.1* for yield. Cytochrome b561, a protein family plays an important role in plant growth, development, and prevention of damage to plants from excess light under drought condition^93^. Recently, the novel MYB-like transcription factor OsMPH1 was identified, where the over expression of OsMPH1 increases plant height and grain yield in rice^72^. SnRK1 (Sucrose-non fermentation1-related protein kinase1) activity plays multiple essential roles in plant growth and in modulating early seedling development after seed germination and in controlling age-dependent leaf senescence^94^. MYB-like transcription factor and SnRK1 underlie the stable QTLs for plant height (*qPH1*), biomass (*qBM1*) and total dry matter (*qTDM1*) on chromosome 1 in our study. Lim *et al*.^53^ identified strong candidate genes underlying *qTln2* for tiller number, which encode receptor-like protein kinase precursor, DUF803 domain containing proteins. A major QTL *qSSL1b* for seedling shoot length was delimited to 80.5 kb region and 16 genes were annotated which includes DUF803 domain containing protein, expressed proteins and candidate region for plant height on chromosome 1^72^. Thus, several candidate genes including F box protein and DUF domain and zinc finger proteins underlie the major effect QTLs for plant height, yield and biomass.

Among 31 significant lines identified in the BILs, 23 also belonged to the CSSL set. Among these 22 lines for DFF and 4 lines for DM were positively significant and 11 were negatively significant for these two traits. Two lines NK41 and NK70 which were negatively significant for flowering days were positively significant for plant height. Plant height and days to flowering were associated in Swarna/*O. nivara* introgression lines^16,95^. In all, 8 lines were positively significant for plant height and 7 of them had a chromosome segment harbouring QTL for plant height at chromosome 1 from *O.nivara*. Five of the lines positively significant for PH were positively significant for BM also. NK61 showed significantly higher value than Swarna for GY and PDP and significantly lower value for DFF and DM. This line harboured alleles from *O.nivara* for GY, PW and PDP at chromosome 8 and for GY, BY, HI, PDP at chromosome 11 but Swarna alleles for DFF and is an important line for fine mapping of major QTLs for these traits.

Among the CSSLs, 4 lines were positively significant over Swarna for days to flowering and maturity and harboured Swarna alleles at all the three QTL loci significantly associated with DFF and DM. Conversely, 11 significantly negative lines identified for flowering days harboured a combination of alleles of *O.nivara* and Swarna except in case of NK79, NK82 and NK88 where alleles at chromosome 3 for DFF and DM were only from *O.nivara*. Secondary mapping populations from crosses involving a QTL-CSSL with recurrent parent or among two CSSLs carrying different QTL segments will help in developing near isogenic lines (NILs), fine mapping and studying QTL **×** QTL interaction^39,67,96^. The set of lines which show significant phenotypic and corresponding genotypic difference for the particular chromosome can be used to create a secondary mapping population for fine mapping of the specific trait.

## Conclusion

A CSSL set of 70 lines of Swarna/ *Oryza nivara* IRGC 81832 with 94.4% genome coverage of *O. nivara* was identified. The 23 significantly different CSSLs identified in this study with wide range of variation for 7 yield traits can be further crossed among themselves for trait pyramiding and to study gene interactions in a largely similar genetic background. The chromosomal region between RM206 and RM144 showed a significant major QTL *qGY11.1* for GY detected consistently across years and average environment. The same locus was mapped as closely linked to BY, GY and PDP in the derived CSSL population also. This is a high priority candidate region for yield improvement. Similarly ten consistent QTLs *qBM1.1, qDFF3.1, qDFF7.1, qDFF12.1, qDM3.1, qDM7.1, qDM12.1, qPH1.2, qNT2.1* and *qTDM11.1* for BM, DFF, DM, PH, NT and TDM were observed over the years and across different mapping techniques such as SMA, ICIM, MET and CSL options. CSSLs with marker defined chromosome segments help in identification of QTLs, underlying genes, and superior lines for any trait of interest and in broadening the genetic base of cultivars.

## Supplementary information

Dataset1.

Dataset 2.

## Data Availability

All data generated or analysed during this study are included in this published article and its Supplementary information files.
