# Supplementary material for: Detecting CSSLs and yield QTLs with additive, epistatic and QTL×environment interaction effects from Oryza sativa × O. nivara IRGC81832 cross
Source: Sci Rep. 2020 May 8;10:7766. doi: 10.1038/s41598-020-64300-0 (PMC7210974; doi:10.1038/s41598-020-64300-0)
Supplement: Supplementary file 1 — Dataset1. [file 41598_2020_64300_MOESM1_ESM.pdf]

Detecting CSSLs and yield QTLs with additive, epistatic and QTL×environment interaction effects from *Oryza sativa* × *O.nivara* IRGC81832 cross

Divya B, Malathi S, Rao YV, Krishnamraju A, Sukumar M, Kavitha B, Sarla N*
ICAR- National Professor Project, ICAR-Indian Institute of Rice Research, Hyderabad, India
*sarla_neelamraju@yahoo.com

Concise Title: CSSLs and consistent yield QTLs from wild introgression lines

Supplementary Material

Supplementary Fig. 1. Frequency distribution of twelve yield traits in Swarna/ *O. nivara* derived backcross introgression lines using adjusted mean of 3 years phenotypic data.

Supplementary Fig. 2. Box plots of twelve yield traits in Swarna/*O. nivara* derived backcross introgression lines in 3 years phenotypic data.

Supplementary Table 1. Details of phenotypic traits of parents and mean range in 90 backcross introgression lines in 2014, 2015 and 2016

Supplementary Table 2. Analysis of variance (ANOVA) for yield traits in the backcross introgression lines

Supplementary Table 3. Test for Normality for yield traits using Shapiro-Wilk method by PBTools v.1.4

Supplementary Table 4. Tests for Homogeneity of variances using Barlette and Levene methods by PBTools v.1.4

Supplementary Table 5. Pooled Analysis of variance(ANOVA) for yield and yield related traits in the backcross introgression lines

Supplementary Table 6. Significant pairwise comparisons of lines compared with control ie. Swarna using phenotypic data of 2014, 2015, 2016

Supplementary Figures

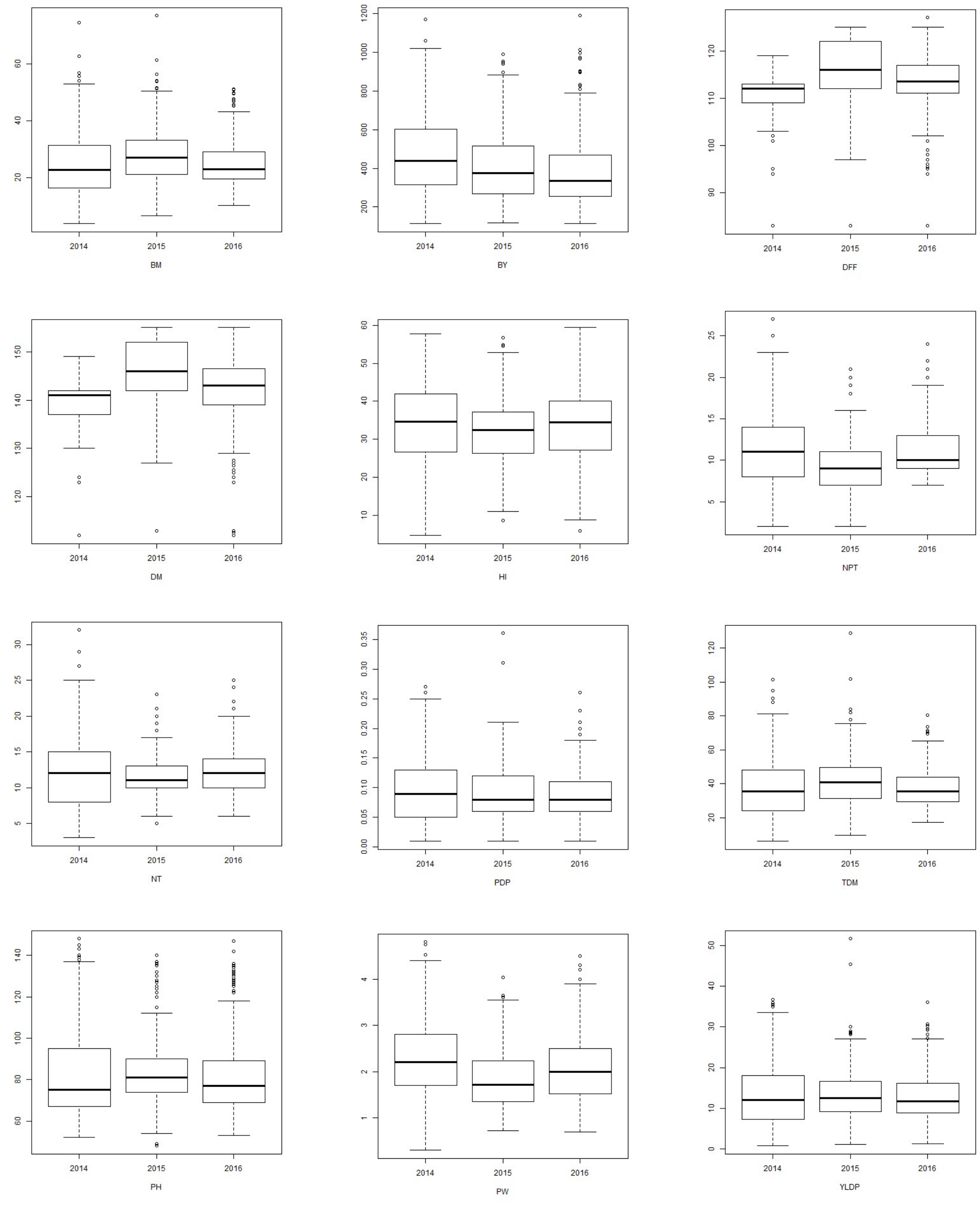

Supplementary Fig. 1 Box plots of 3 years phenotypic data of twelve yield traits in Swarna/*O. nivara* derived backcross introgression.

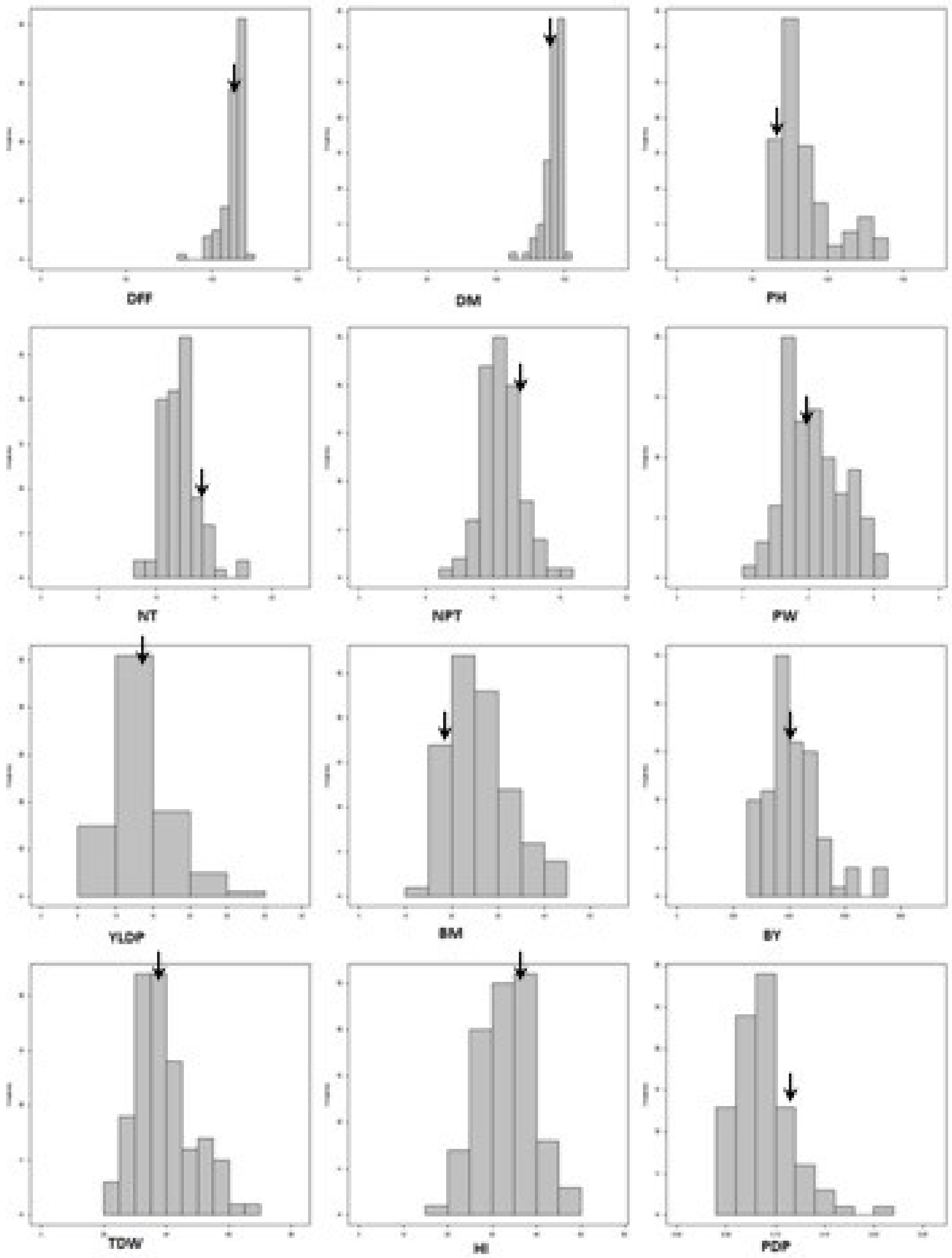

Supplementary Fig. 2 Frequency distribution of twelve yield traits in Swarna/*O. nivara* derived backcross introgression lines using adjusted mean of 3 years phenotypic data.

Supplementary Table 1. Details of phenotypic traits of parents and mean range in 90 backcross introgression lines in 2014, 2015 and 2016

Variable	ENV	Min	Max	Mean	StdDev	SE_Mean	CV	Skewness	Kurtosis
DFF	2014	83.00	119.00	110.33	5.72	0.35	5.19	-2.12	5.91
DFF	2015	83.00	125.00	115.53	7.53	0.46	6.52	-1.51	3.22
DFF	2016	83.00	127.00	112.98	6.97	0.42	6.17	-1.33	3.35
DM	2014	112.00	149.00	139.02	5.86	0.36	4.21	-1.78	4.84
DM	2015	113.00	155.00	145.51	7.53	0.46	5.18	-1.50	3.21
DM	2016	112.00	155.00	142.26	7.13	0.43	5.01	-1.18	2.76
PH	2014	52.00	148.00	83.97	23.27	1.42	27.72	1.08	0.12
PH	2015	48.00	140.00	85.05	18.00	1.10	21.16	1.20	1.25
PH	2016	53.00	147.00	83.90	21.56	1.31	25.69	1.23	0.51
NT	2014	3.00	32.00	11.86	4.77	0.29	40.24	0.51	0.76
NT	2015	5.00	23.00	11.46	2.96	0.18	25.81	0.43	0.78
NT	2016	6.00	25.00	12.83	3.67	0.22	28.63	1.24	1.15
NPT	2014	2.00	27.00	11.20	4.49	0.27	40.09	0.38	0.15
NPT	2015	2.00	21.00	9.49	3.18	0.19	33.48	0.41	0.72
NPT	2016	7.00	24.00	11.41	3.30	0.20	28.93	1.47	2.10
PW	2014	0.30	4.80	2.32	0.81	0.05	35.08	0.61	0.26
PW	2015	0.72	4.03	1.85	0.61	0.04	32.74	0.83	0.42
PW	2016	0.70	4.50	2.07	0.69	0.04	33.46	0.52	0.52
GY	2014	0.82	36.73	13.15	7.73	0.47	58.81	0.75	0.30
GY	2015	1.20	51.60	13.48	6.40	0.39	47.52	1.63	5.98
GY	2016	1.30	36.00	12.95	5.75	0.35	44.40	1.03	1.17
BM	2014	3.80	74.50	24.63	12.04	0.73	48.88	0.84	0.77
BM	2015	6.50	77.00	28.47	10.72	0.65	37.65	0.83	1.19
BM	2016	10.10	51.20	24.78	8.10	0.49	32.70	0.94	0.86
BY	2014	114.02	1169.19	461.76	202.95	12.35	43.95	0.57	0.16
BY	2015	117.44	990.00	405.27	181.03	11.02	44.67	0.99	0.69
BY	2016	115.50	1188.00	388.35	182.36	11.10	46.96	1.41	2.31
TDM	2014	6.05	101.43	37.78	18.11	1.10	47.93	0.74	0.43
TDM	2015	9.70	128.60	41.95	15.39	0.94	36.70	1.15	3.67
TDM	2016	17.10	80.20	37.73	11.46	0.70	30.36	0.86	0.60
HI	2014	4.68	57.73	34.04	11.16	0.68	32.79	-0.14	-0.46
HI	2015	8.63	56.77	31.99	8.33	0.51	26.03	0.14	0.15
HI	2016	5.88	59.37	34.08	9.56	0.58	28.05	-0.05	-0.06
PDP	2014	0.01	0.27	0.10	0.06	0.00	60.17	0.79	0.35
PDP	2015	0.01	0.36	0.09	0.05	0.00	48.65	1.61	5.51
PDP	2016	0.01	0.26	0.09	0.04	0.00	45.87	1.04	1.16

Supplementary Table 2. Analysis of variance (ANOVA) for yield traits in backcross introgression lines

Trait Name	Env Name	DF_ Error	DF_ Block	DF_ Geno	MS_ Error	MS_ Block	MS_ Geno	VA_ Error	VA_ Block	VA_ Geno
DFF	2014	180	2	90	0.00	0.00	97.91	0.00	0.00	32.64
DFF	2015	180	2	90	0.00	0.00	169.65	0.00	0.00	56.55
DFF	2016	179	2	90	4.75	649.32	120.19	4.75	7.11	38.62
DM	2014	180	2	90	0.00	0.00	102.90	0.00	0.00	34.30
DM	2015	180	2	90	0.08	0.08	169.55	0.08	0.00	56.49
DM	2016	179	2	90	5.32	973.04	118.90	5.32	10.67	38.00
PH	2014	180	2	90	33.87	50.08	1550.18	33.87	0.18	505.44
PH	2015	180	2	90	17.08	5.31	934.34	17.08	0.00	305.75
PH	2016	179	2	90	27.68	31.35	1332.43	27.68	0.04	436.52
NT	2014	180	2	90	22.49	32.12	25.74	22.49	0.11	1.08
NT	2015	180	2	90	6.81	2.81	12.66	6.81	0.00	1.95
NT	2016	179	2	90	9.41	38.67	20.83	9.41	0.32	3.82
NPT	2014	180	2	90	19.34	26.26	22.71	19.34	0.08	1.12
NPT	2015	180	2	90	7.78	3.49	14.92	7.78	0.00	2.38
NPT	2016	179	2	90	8.42	22.56	15.40	8.42	0.16	2.33
PW	2014	180	2	90	0.39	0.68	1.26	0.39	0.00	0.29
PW	2015	180	2	90	0.15	0.23	0.79	0.15	0.00	0.21
PW	2016	179	2	90	0.13	0.25	1.19	0.13	0.00	0.35
GY	2014	180	2	90	37.59	80.13	104.78	37.59	0.47	22.40
GY	2015	180	2	90	23.73	8.11	75.25	23.73	0.00	17.17
GY	2016	179	2	90	14.96	7.77	66.73	14.96	0.00	17.32
BM	2014	180	2	90	95.89	48.16	241.25	95.89	0.00	48.45
BM	2015	180	2	90	68.10	82.25	208.85	68.10	0.16	46.92
BM	2016	179	2	90	20.80	12.60	154.77	20.80	0.00	44.82
BY	2014	180	2	90	30314.02	157276.30	61825.99	30314.02	1395.19	10503.99
BY	2015	180	2	90	17114.41	487419.75	53642.13	17114.41	5168.19	12175.91
BY	2016	179	2	90	15245.49	461927.41	56065.21	15245.49	4926.64	13656.60
TDM	2014	180	2	90	228.22	193.94	523.29	228.22	0.00	98.36
TDM	2015	180	2	90	141.91	94.26	424.41	141.91	0.00	94.17
TDM	2016	179	2	90	39.43	12.17	311.55	39.43	0.00	91.04
HI	2014	180	2	90	50.76	218.49	272.94	50.76	1.84	74.06
HI	2015	180	2	90	40.47	13.54	133.73	40.47	0.00	31.09
HI	2016	179	2	90	47.26	52.93	174.43	47.26	0.06	42.54
PDP	2014	180	2	90	0.00	0.00	0.01	0.00	0.00	0.00
PDP	2015	180	2	90	0.00	0.00	0.00	0.00	0.00	0.00
PDP	2016	179	2	90	0.00	0.00	0.00	0.00	0.00	0.00

Supplementary Table 3. Test for Normality for yield traits based on environment grouping Shapiro-Wilk method in PBTools v.1.4

Year	Variable	W Value	Pr(< W)
2014	DFF	0.795	0.000
2015	DFF	0.866	0.000
2016	DFF	0.907	0.000
2014	DM	0.848	0.000
2015	DM	0.867	0.000
2016	DM	0.926	0.000
2014	PH	0.869	0.000
2015	PH	0.890	0.000
2016	PH	0.846	0.000
2014	NT	0.974	0.000
2015	NT	0.976	0.000
2016	NT	0.870	0.000
2014	NPT	0.981	0.001
2015	NPT	0.978	0.000
2016	NPT	0.855	0.000
2014	PW	0.969	0.000
2015	PW	0.947	0.000
2016	PW	0.969	0.000
2014	GY	0.956	0.000
2015	GY	0.904	0.000
2016	GY	0.937	0.000
2014	BM	0.956	0.000
2015	BM	0.959	0.000
2016	BM	0.944	0.000
2014	BY	0.974	0.000
2015	BY	0.931	0.000
2016	BY	0.889	0.000
2014	TDM	0.963	0.000
2015	TDM	0.945	0.000
2016	TDM	0.951	0.000
2014	HI	0.992	0.149
2015	HI	0.995	0.465
2016	HI	0.997	0.859
2014	PDP	0.949	0.000
2015	PDP	0.897	0.000
2016	PDP	0.930	0.000

Supplementary Table 4. Tests for Homogeneity of Variances based on environment grouping Barlette and Levene method in PBTools v.1.4

Variable	DF	Statistic	Value	Prob	p Value
DFF	2	Chisq	20.61	Pr(>Chisq)	0.000
DFF	2	F	8.43	Pr(>F)	0.000
DM	2	Chisq	17.84	Pr(>Chisq)	0.000
DM	2	F	5.38	Pr(>F)	0.000
PH	2	Chisq	17.99	Pr(>Chisq)	0.000
PH	2	F	6.65	Pr(>F)	0.000
NT	2	Chisq	61.17	Pr(>Chisq)	0.000
NT	2	F	24.41	Pr(>F)	0.000
NPT	2	Chisq	40.72	Pr(>Chisq)	0.000
NPT	2	F	21.59	Pr(>F)	0.000
PW	2	Chisq	23.3	Pr(>Chisq)	0.000
PW	2	F	8.27	Pr(>F)	0.000
GY	2	Chisq	24.57	Pr(>Chisq)	0.000
GY	2	F	10.59	Pr(>F)	0.000
BM	2	Chisq	41.76	Pr(>Chisq)	0.000
BM	2	F	13.66	Pr(>F)	0.000
BY	2	Chisq	4.5	Pr(>Chisq)	0.110
BY	2	F	3.65	Pr(>F)	0.030
TDM	2	Chisq	54.55	Pr(>Chisq)	0.000
TDM	2	F	17.28	Pr(>F)	0.000
HI	2	Chisq	23.05	Pr(>Chisq)	0.000
HI	2	F	13.26	Pr(>F)	0.000
PDP	2	Chisq	29.26	Pr(>Chisq)	0.000
PDP	2	F	13.01	Pr(>F)	0.000

Supplementary Table 5. Pooled Analysis of variance (ANOVA) for yield and yield related traits in 90 backcross introgression lines

Trait Name	Environment Name	DF_ Error	DF_ Block	DF_ Gen	DF _Env	DF_ GbyE	MS_ Error	MS_ Block	MS_ Geno	MS_ Env	MS_ GbyE	VA_ Error	VA_ Block	VA_ Geno	VA_ Env	VA_ GbyE
DFF	Combined Env	539	6	90	2	180	1.58	216.46	358.92	1873.34	14.42	1.58	2.36	39.75	6.86	4.29
DM	Combined Env	539	6	90	2	180	1.79	324.40	359.91	2927.59	15.72	1.79	3.55	39.84	10.73	4.65
PH	Combined Env	539	6	90	2	180	26.21	28.91	3256.64	108.55	280.15	26.21	0.03	359.38	0.30	84.75
NT	Combined Env	539	6	90	2	180	12.90	24.51	21.89	133.33	18.69	12.90	0.13	1.00	0.44	1.93
NPT	Combined Env	539	6	90	2	180	11.85	17.43	19.75	301.70	16.64	11.85	0.06	0.88	1.06	1.60
PW	Combined Env	539	6	90	2	180	0.22	0.39	1.86	15.96	0.69	0.22	0.00	0.18	0.06	0.15
GY	Combined Env	539	6	90	2	180	25.44	32.00	135.53	24.94	55.62	25.44	0.07	12.25	0.00	10.07
BM	Combined Env	539	6	90	2	180	61.67	47.67	399.19	1221.35	102.85	61.67	0.00	37.55	4.25	13.74
BY	Combined Env	539	6	90	2	180	20906.56	368897.16	85429.15	436336.97	43034.90	20906.56	3828.75	7177.94	1523.58	7385.13
TDM	Combined Env	539	6	90	2	180	136.67	100.10	789.93	1539.88	234.75	136.67	0.00	72.67	5.15	32.73
HI	Combined Env	539	6	90	2	180	46.19	95.04	311.77	337.63	134.58	46.19	0.54	29.55	1.07	29.50
PDP	Combined Env	539	6	90	2	180	0.00	0.00	0.01	0.00	0.00	0.00	0.00	0.00	0.00	0.00

Supplementary Table 6. Significant pairwise comparisons of backcross introgression lines compared with Swarna using phenotypic data of 2014, 2015, 2016, Only significantly different lines are shown

**Line #**	DFF	DM	PH	GY	BM	TDM	HI	PDP
NK22			*		*			
NK27			*		*		#	
NK28			*		*		#	
NK29		*	*					
NK30	*	*						
NK31	*	*						
NK32							#	
NK33		*						
NK40			*					
NK41	#	#	*					
NK44	#	#						
NK45			*					
NK46	#	#						
NK58			*		*	*		
NK59	*	*						
NK60	*	*						
NK61	#	#		*				*
NK64	*	*						
NK67	#	#						
NK68			*		*			
NK70	#	#	*					
NK71							#	
NK72	#		*					
NK74	#	#						
NK77	*	*						
NK78			*		*			
NK79	#	#						
NK82	#	#						
NK83			*		*	*		
NK88	#	#						
NK90	#	#						
Total number of lines significantly different from Swarna						31		
*Lines with significantly higher values for a trait						21		
#Lines with significantly lower values for a trait						16		

Trait	DFF	DM	PH	NT	NT	PW	GY	BM	BY	HI	PDP
positive	6	8	13	0	0	0	1	7	2	0	1
negative	12	11	0	0	0	0	0	0	0	4	0

*-Positive=ILs with significantly higher value; #-negative= ILs with significantly lower value than Swarna
